# Forensic stature estimation: a systematic review of the correlation between footprints and individual height

**DOI:** 10.1007/s12024-025-01110-8

**Published:** 2025-10-30

**Authors:** Sara García-Mora, Laura Regife-Fernández, Natalia Tovaruela-Carrión, María José Manfredi Márquez, María Vázquez-Castro, Juan Alvárez-Cordero, Rosario Correa-Rodriguez, Aurora Castro-Méndez

**Affiliations:** 1 Private Podiatry Clinic in Madrid, Madrid, 28001 Spain; 2 Departamento de Podología, Facultad de Enfermería, Fisioterapia y Podología, Grupo de Investigación DS-30 Bases biomédicas del pie que afectan al apoyo y la marcha, Instituto de Biomedicina de Sevilla, IBiS, Universidad de Sevilla, Sevilla, 41009 Spain; 3 Private Podiatry, Pilar St. Alcala de Guadaira, Sevilla, 41500 Spain; 4 Luis Montoto ST. Sevilla, Sevilla, 41018 Spain; 5 Departamento de Podología, Facultad de Enfermería, Fisioterapia y Podología. Universidad de Sevilla, Sevilla, Spain

**Keywords:** Foot, Body height, Footprint, Length, Forensic medicine

## Abstract

Anthropometric identification is crucial in legal contexts, where height is a relevant distinguishing characteristic. The estimation of height through plantar impressions is a tool used in forensic podiatric examinations, and it is relevant to be familiar with the most suitable techniques for correlating the length of the footprints with height. This systematic review aims to evaluate forensic identification methods by analysing inked footprint collection and measurement techniques, their correlation with height, and the available scientific evidence. A systematic review of the available literature was conducted in the following databases: PubMed, Embase, Scopus, Web of Science, CINAHL, and Dialnet. Descriptive observational studies linking the length of plantar impressions of healthy adults of both sexes with their height were included. Seven studies published between 2012 and 2020 were selected. All studies showed statistically significant correlations between height and plantar impression measurements, analyzed using Pearson´s correlation coefficient. The inked footprint method was the most widely used and demonstrated high reliability. Additionally, techniques based on foot contours and pedigraphs also showed strong correlations with height and foot length. However, the scientific evidence on this topic is limited, highlighting the need for further research to optimise these techniques and enhance their validity for forensic applications.

**Supplementary information:**

The online version contains supplementary material available at 10.1007/s12024-025-01110-8.

## Introduction

Forensic podiatry is a specialized discipline that applies podiatric knowledge to legal investigations, offering valuable support in scenarios such as crime scene analysis, identification of human remains, and interpretation of foot-related evidence [1]. This field requires a multidisciplinary approach in which the podiatrist contributes to the identification of individuals, both living and deceased, through the examination of barefoot footprints, footwear impressions, or images obtained from closed-circuit television systems [2, 3].

Among the various biological markers used for identification, stature estimation holds particular forensic importance. It serves as a key element in constructing the biological profile of unidentified remains, especially in cases where the body is incomplete or severely decomposed. One of the most critical contributions of forensic podiatry is the development of a biological profile, which includes sex, age, and height estimation. These parameters are essential in narrowing down the pool of possible victims or suspects, particularly in cases involving incomplete remains or isolated body parts [4]. Among these variables, height is especially relevant in forensic contexts because it is a stable anatomical marker that can assist in both identification and exclusion processes [5].

Various anthropometric techniques have been developed over the years to estimate height, with long bone measurement being the most commonly used due to its reliability and standardization in osteological analysis. However, in many forensic cases, especially those involving mass disasters, dismemberment, or advanced decomposition, complete long bones may not be available. This limitation has prompted researchers to explore alternative body parts such as hands, vertebrae, and feet as potential proxies for stature estimation. Anthropometry offers various means to estimate height, such as measuring long bones, hand dimensions, or foot dimensions, particularly the length of the foot, which has proven to be a reliable predictor [5, 6]. In forensic cases where full skeletal remains are unavailable, footprints or partial body remains can still yield useful information for height estimation, making foot length a valuable proxy [7, 8].

Footprints, complete or partial, 2D or 3D, are considered unique and non-transferable, which makes them suitable for personal identification. Furthermore, foot structure can reveal congenital deformities, pathological conditions, or surgical history, all of which contribute to a more comprehensive profile [4, 5]. However, it is important to consider variability across populations, where racial, ethnic, and regional differences can affect anthropometric relationships and regression models [9].

Several studies have attempted to correlate foot length or footprint dimensions with height, employing various methodologies including inked footprint techniques, foot contours, and pedigraphs [10–13]. These tools facilitate the construction of regression equations that are often specific to the population studied and depend on the technique used to record the footprint [14]. While these methods differ in terms of accuracy, accessibility, and application, the correlation between foot length and height remains a promising alternative due to the relative ease of obtaining footprints in both clinical and field settings. Nevertheless, inconsistencies in methodology and lack of standardization limit the comparability and generalizability of current findings.

Therefore, the aim of this review is to analyze and compare the methodologies most frequently used to assess the correlation between foot length and height, specifically focusing on whether the inked footprint technique is more commonly used and more effective than the pedigraph or foot contour methods. This will help to identify the most suitable technique for forensic application and highlight current gaps in the literature regarding standardized methodologies.

## Methods

### Search approach

To performed this systematic review, we conducted an exhaustive search of the scientific literature in the databases PubMed, Embase, Scopus, Web of Science, CINAHL, and Dialnet Plus. We also explored the gray literature in specialized repositories and Google Scholar to minimize bias. The review was conducted in accordance with the Preferred Reporting Items for Systematic Reviews (PRISMA) guidelines. The systematic review was registered in the Open Science Framework (OSF) under the name: “Correlation between footprint and subjects’ height. A systematic review” Available at: https://archive.org/details/osf-registrations-3grdj-v1.

The research question followed the PICOS criteria (P-Patients: healthy adult population; I- Intervention: Measurement of the footprint length using the inked footprint technique and its correlation with the subject’s height; C- Comparison with paper pedigraphy and with a paper footprint outline; and O- Outcomes: Which is the most widely used footprint taking and measuring technique, which is most correlated and has the most scientific evidence).

To conduct the literature search, indexed descriptors and free text words related to the research objective were used. The selected terms were linked with Boolean operators (AND, OR, and NOT). Three search strategies were obtained in the different databases: (“foot length”) AND (“body height”); (“foot measurement”) AND (“body height”) OR (“foot measurement”) AND (“stature estimation”)); (“foot measurement”) AND (“body height”) AND (“forensic science”) OR (“reliability foot measurement”) AND (“body height”) AND (“forensic science”)). The search was carried out from January to March 2023.

### Article selection

After eliminating duplicate articles, two independent reviewers reviewed the articles by title and abstract and proceeded to eliminate those that did not meet the inclusion criteria. If these two reviewers disagreed on the choice, a third reviewer intervened to reach a consensus.

The following inclusion and exclusion criteria were taken into account when selecting the articles. Inclusion Criteria: (1) documents published in the last 20 years (1993–2023), (2) Spanish or English language, healthy adult population of both sexes, (3) type of study: observational, descriptive, cross-sectional, experimental, systematic reviews, meta-analysis, randomized and non-randomized clinical studies, (4) studies in which the height of the subjects is estimated by taking and measuring the length, exclusively, of the footprints of both feet and (5) studies with living subjects with intact feet and without macroscopic deformity. On the other hand, the following were excluded: (1) studies with subjects with foot or spinal disease or deformity, pregnant women, orthopedic problems, physical impairments, growth-related diseases, and physical disabilities; (2) studies with cadavers; (3) studies that estimated subjects’ height by measuring: length of the metatarsals, hands, and feet simultaneously, a single foot, stride length during walking, through shoe measurement, or by measuring more than one anthropometric measurement of the foot (width, scaphoid height, etc.) or the lower limb (hip, leg); and (4) studies where the sample included only one sex.

### Data and study features extraction

The analysis involved extracting data from each study. From each of the selected articles, the following information was extracted: author(s) name, study title, year of publication, type of design, objectives, method and duration of the study, footprint length measurement technique, population, results, conclusions, and study quality.

### Quality assessment

The studies included in this systematic review were observational, descriptive, and cross-sectional in design [15–17]. To assess the quality of the scientific evidence, we applied the classification system of the Canadian Task Force on the Periodic Health Examination [18], which categorizes evidence according to study design and methodological strength.

According to this scale, all included studies were classified as Level III evidence, which corresponds to evidence obtained from non-experimental, descriptive studies such as comparative, correlation, or case-control studies. This rating is consistent with the methodological characteristics of the included articles, which did not involve randomized interventions but rather correlation analyses between footprint dimensions and height in population samples.

Each study was evaluated for key methodological components, including: clarity of inclusion and exclusion criteria, sample size and demographic description, footprint measurement techniques, height measurement protocols, use of appropriate statistical analyses (correlation coefficients, regression models), reporting of results and statistical significance.

Although none of the studies involved blinding or randomization (as expected in this type of research), they were generally methodologically sound for their observational design. No study was excluded based on quality, as all met the minimum criteria for descriptive observational research and addressed the review’s PICO question adequately.

## Results

### Search yield

The bibliographic search using the different strategies yielded the following: PubMed produced 172, 90, and 42 articles in strategies one, two, and three, respectively. Embase retrieved 122, 91, and 45 results. Scopus yielded 2,530, 4,452, and 522 results. Web of Science identified 429, 388, and 20 articles. CINAHL provided 110, 91, and 3 results, while Dialnet only yielded 16 articles in the first strategy and none in the second and third.

**Fig. 1:**
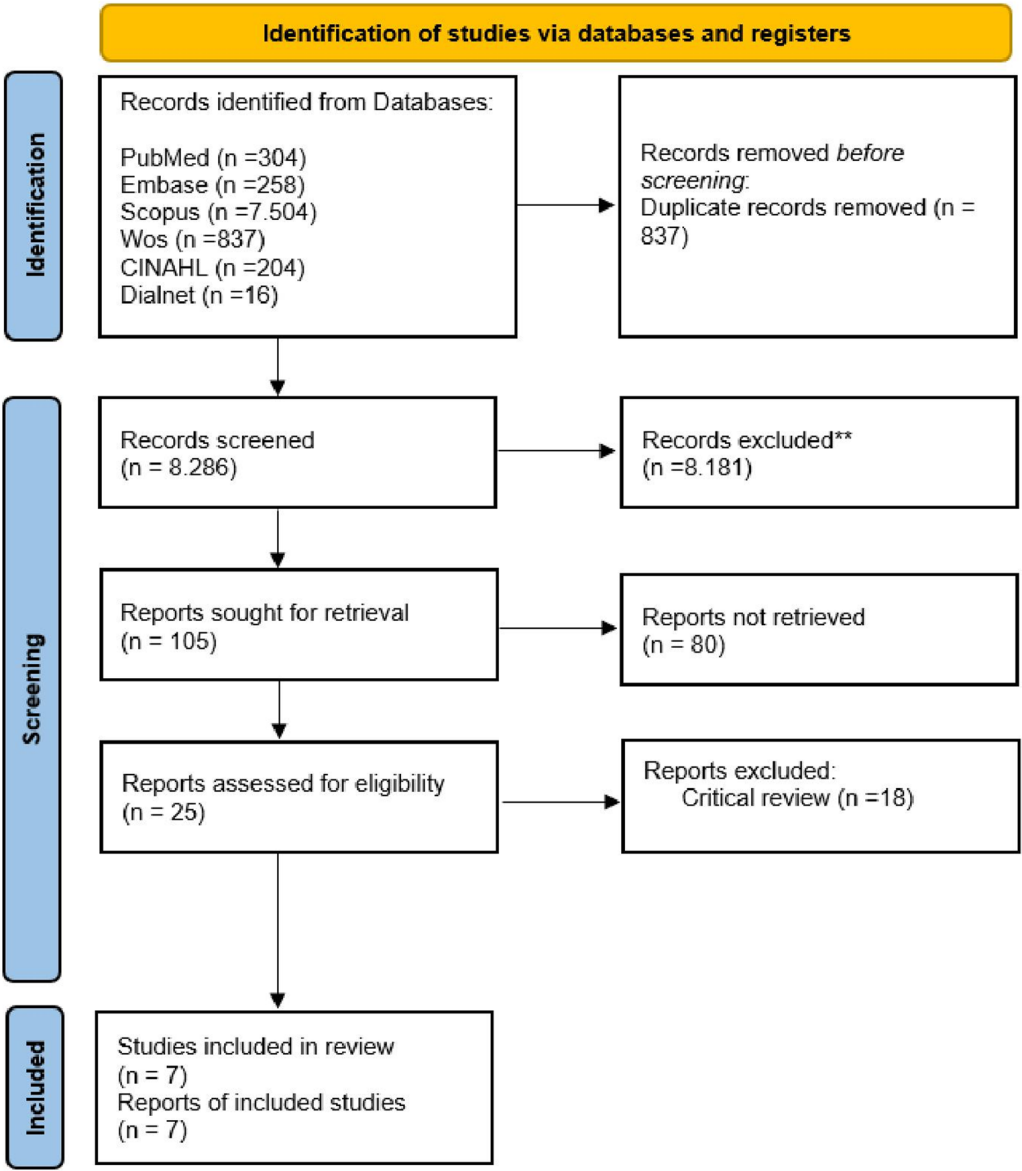
PRISMA 2020 flow diagram

After removing duplicates and screening titles via Zotero, 105 articles were retained. Abstract and full-text screening based on inclusion criteria narrowed the selection to 25. Ultimately, seven studies met the PICO requirements and were included in the review. Figure 1 shows the PRISMA flow diagram.

### Study characteristics and grouped results

The aim across all included studies was to evaluate the correlation between individuals’ height and various footprint length measurements, using different techniques for footprint acquisition and measurement protocols.

#### Footprint acquisition techniques

Three primary methods were employed:

- Inked footprint technique (ink-based), used in four studies [10–12, 19], involved applying ink to the sole and stepping on paper to capture the footprint.
- Foot outline (contour) method, used in two studies [4, 20], involved placing the foot on paper and tracing its perimeter with a pencil.
- Pedigraphy, applied in two studies [19, 21], used pressure-based contact on paper to transfer the foot’s shape.

The inked method was the most commonly used, offering clear 2D representations. However, no study directly compared the effectiveness of each method in terms of correlation strength or accuracy.

#### Footprint measurement approaches

Two types of measurements were documented:

- Single-point measurement: From the heel to the tip of the longest toe [11, 12]. Simple and fast, this approach was used in studies using the ink or dye-based method.
- Multi-point measurement: From the heel to the tips of all five toes [4, 10, 22–24]. These studies recorded five measurements per foot, typically for both feet, enhancing precision and analysis.

Some studies also included foot circumference lengths [20, 22], providing complementary anthropometric data.

#### Statistical analyses applied

All studies used linear regression analysis to assess the relationship between footprint length and height. Some included multiple regression with additional variables such as foot circumference [22], enhancing predictive capacity.

Most studies reported statistically significant Pearson correlation coefficients (r), with values ranging from moderate (*r* ≈ 0.6–0.7) to high (*r* ≈ 0.85–0.88) [4, 10, 22].

One study [21] used an alternative ratio index, calculating mean ratios of footprint length to height and applying an error margin of ± 2 cm to estimate individual height. Although less common, this method yielded reliable estimates within an acceptable error range.

#### Sex-based comparisons

Studies that analyzed data by sex (male/female) found that pooled data often yielded higher correlation coefficients than sex-disaggregated data [4, 10, 21, 22]. Nonetheless, results within each sex group were still statistically significant, and correlation remained strong in both genders.

This suggests that in forensic cases where sex is unknown, using pooled regression models may be a valid approach for estimating stature from footprints.

#### Level of scientific evidence

According to the Canadian Task Force on the Periodic Health Examination, all included studies were classified as Level III evidence [18], due to their cross-sectional and observational nature. No randomized or controlled trials were identified.

#### Forensic implications

All studies concluded that footprint length can serve as a reliable proxy for estimating stature, particularly in forensic scenarios involving incomplete remains, dismembered bodies, or isolated footprints at crime scenes.

The inked footprint method was the most practical and widely used, while the heel-to-longest-toe measurement was the most consistent predictor across techniques. Multi-point methods provided enhanced data but were more time-consuming.

The findings suggest that in both forensic and anthropological contexts, regression models derived from footprint dimensions are effective, provided they are used within the studied population framework. The results are showed in Table 1.

**Table 1:** Evidence Table. Data extraction from selected Studies. Correlation between subjects’ height and plantar footprints

Author/s, Years	Study title	Design type	Objective	Method and duration	Technique for Measuring the Length of Plantar Footprints	Sample size and age	Level of evidence	Results	Conclusions
Kanchan et al., 2012	*Analysis of footprint and its parts for stature estimation in Indian population.*	Cross-sectional descriptive observational study	Estimate the height of subjects by measuring the length of their footprints.	• Height (cm): Vallois Technique • Footprints (Left, Right): Glass plate, Roller, Black ink, White paper	Five measurements in cm from: • Tthe midpoint of the posterior heel to the most anterior point of each toe (T1, T2, T3, T4, T5)	*N* = 100 (200 footprints) Healthy young adults aged 20–25 years Mangalore, India	III	Correlation coefficients are statistically significant (*p* < 0.001) for the relationship between height and various footprint length measurements.	There is a strong correlation between different footprint measurements and height. An individual’s height can be estimated from their footprints.
Imran et al., 2013	*Estimation of stature from footprint length.*	Cross-sectional descriptive observational study	Estimate the height of subjects by measuring of their footprints.	• Fixed time: 14:00 to 16:00 h • Height (cm): measuring tape • Footprints (Left, Right): Glass plate, Roller, Black ink, White paper	One measurement in cm from: • The outermost margin of the heel to the tip of the longest toe in the footprint.	*N* = 200 (400 footprints) Healthy young students Gulbarga region, India	III	The prediction of height based on footprint length is possible, as the difference in the correlation coefficient is statistically significant (*p* < 0.01).	Height can be predicted by measuring the length of the right and left footprints.
Moorthy et al., 2014	*Estimation of stature from footprint and foot outline measurements in Malaysian Chinese.*	Cross-sectional descriptive observational study	Estimate height based on bilateral footprints and foot outlines	• Fixed afternoon schedule • Height (cm): height measuring device • Footprints (Left, Right): Inkless Shoe Print Kit/Pedigraphy	Five measurements in cm in pedigraphy from: • Midpoint of the outermost margin of the heel. • Anterior part of all toes. The foot outline drawing was included.	*N* = 200 (400 footprints) Healthy young adults aged 18–55 years Malasia	III	High significant positive correlation between height, footprint length, and foot outline length.	The regression equations derived from the sample can be used to estimate height.
Kumar et al., 2014	*Evaluation of stature from dimensions of footprints.*	Cross-sectional descriptive observational study	Estimate the height of subjects by measuring the length of their footprints.	December 2013 to May 2014. • Height (cm): Anthropometer • Footprints (Left, Right): “Inkless footprint kit LE 25 with alpha thermal paper” -Pedigraphy.	Five measurements in cm from: • The most posterior point of the calcaneus to the most distal points of the five toes.	*N* = 200 (400 footprints) Healthy young adults aged 20–25 years India	III	Height estimation based on the maximum footprint length was highly accurate, with an error margin of only ± 2 cm.	A linear correlation exists between footprint length and an individual’s height.
Hairunnisa et al., 2016	*Stature Estimation from Foot Outline Measurements in Adult Lun Bawang Ethnics of East Malaysia by Regression Analysis.*	Cross-sectional descriptive observational study	Estimate height based on bilateral foot contours and generate population-specific regression equations.	Fixed time: Night. • Height (cm): Portable body measurement device • Footprints (Left, Right): White paper, pencil.	5 measurements in cm from: • Midpoint of the most prominent part of the heel. • Anterior points of all toes.	*N* = 230 (460 footprints) Healthy young adults aged 18–84 years Malasia	III	They show a significant positive correlation between height and foot outline length. They provide regression equations for height estimation based on foot outline measurements.	The regression equation derived from the sample can be used to estimate height when the subject’s sex is unknown.
Nikkimor et al., 2018	*Estimation of stature from foot outline 3(D) anthropometry among Kagay-Anons in Philippines.*	Cross-sectional descriptive observational study	Estimate height based on foot contour measurements.	1 year and 1 month. • Height (cm): Portable stadiometer • Footprints (Left, Right): White paper, pencil.	5 measurements in cm from: • Midpoint of the outermost part of the heel. • Most anterior point of each toe.	*N* = 400 (800 footprints) Healthy young adults over 18 years old. Filipines	III	There is a strong positive correlation between foot outline length and height.	Regression equations are developed to estimate height based on the foot outline anthropometry of the Kagay-anon population in the Philippines.
Janarthanan et al., 2020	*Estimation of stature from footprint length.*	Cross-sectional descriptive observational study	Estimate the height of subjects by measuring the length of their footprints.	1 year and 1 month. • Height (cm): Height measuring device • Footprints (Left, Right): Glass plate, Roller, Black ink, White paper	One measurement in cm from: • The outermost edge of the heel to the tip of the longest toe of the footprint.	*N* = 100 Healthy young students aged 18–22 years India	III	A significant correlation was found between the right footprint length and height (*r* = 0.761) and the left footprint length and height (*r* = 0.747). The difference in the correlation coefficient is statistically significant (*p* = 0.000).	The formula established in this study does not provide a 100% accurate result, but it can be used to estimate an approximate height from a person’s footprint.

##  Discussion

Forensic podiatry applies podiatric expertise in legal investigations, allowing forensic podiatrists to analyze foot-related evidence at crime scenes and contribute expert reports on related legal issues [1, 3]. The use of reliable tools for footprint collection and measurement is crucial for identifying individuals based on their footprints, which is why this systematic review focuses on comparing the most commonly used techniques in forensic practice to estimate height from footprints.

Although limited in number, the studies analyzed consistently aim to estimate stature by measuring footprint length within specific populations. The three primary footprinting methods identified are the inked footprint method [10–12, 21], where the subject’s inked foot is pressed onto white paper; the foot outline technique [4, 25], which involves tracing the foot on paper; and pedigraphy [21, 26], where pressure creates a footprint on paper. Among these, the inked footprint method is the most frequently employed to predict height, yet no study explicitly asserts it has superior statistical correlation compared to the others, despite all reporting high correlation coefficients. The scientific evidence from these studies is rated at quality level III according to the Canadian Task Force on the Periodic Health Examination [18], reflecting the need for further robust research.

Regarding measurement protocols, most studies use either five specific length measurements from the heel to each toe [4, 19–21] or a single measurement from the heel to the longest toe [11, 12]. Literature supports heel-to-toe length as the most reliable foot measurement for estimating stature, which aligns with the prevalence of the heel-to-five-toe approach in the included studies [27–29].

This correlation between footprint length and height is further supported by research outside this review. For example, Kanchan et al. (2010) and Krishan (2008) found strong correlations (*r* >0.7) between foot length and stature in North Indian populations using ink-based and outline methods, respectively [27, 29]. Similarly, Hemy et al. (2013) demonstrated that footprint dimensions reliably predict stature in a Western Australian cohort [2]. These findings reinforce the consistency of footprint-based height estimation across diverse ethnic and geographic populations.

It is relevant the fact that sex-matched samples show higher Pearson correlation coefficients (r) compared to independent male or female groups [4, 10, 21, 22]. However, correlations remain strong and positive within sex-specific groups. From a forensic standpoint, when the sex of an individual from a footprint is unknown, regression models that do not differentiate by sex appear most appropriate, as described in the literature [22].

Most studies utilize linear or multiple regression analyses to estimate height from footprint measurements, with multiple regression often providing greater predictive accuracy [22, 28]. One study [21] uniquely applies a ratio index rather than correlation coefficients to demonstrate the relationship between footprint length and stature. Collectively, these studies report statistically significant correlations (*p* < 0.005) and strong positive relationships (*r* >0) between footprint length and height.

From a practical forensic perspective, these findings underscore that footprints recovered at crime scenes, whether partial or complete, can serve as reliable indicators of a suspect’s height—particularly when population-specific regression equations are applied. This has clear relevance in cases involving unidentified remains, dismemberment, or mass disasters, where other identification methods may be limited. Clinically, footprint-based height estimation might also support growth assessments in pediatric populations or aid biomechanical evaluations, though further validation in such contexts is warranted.

While the results are promising, it is critical to recognize that the applicability of these findings is largely population-specific, limiting direct extrapolation to other groups. To enhance generalizability and forensic utility, future research should prioritize multicentric studies with diverse populations using standardized measurement protocols, thereby enabling meta-analytic approaches and stronger evidence synthesis.

### Limitations

The main limitation found in this systematic review was the ethnic origin of all the selected studies. Most were conducted in Asia, in regions of India such as Mangalore, Gulbarga, Chennai, Bangalore; eastern Malaysia; and the Philippines. Therefore, the data are not easily extrapolated to other populations. Likewise, no studies on legal and forensic podiatry or the correlation between height and footprint length have been conducted in Spain. Another limitation was the inability to assess the risk of bias presented by the seven articles included in this study, as there are no specific risk of bias scales for descriptive observational studies. Finally, only descriptive observational studies were found during the literature search. No other types of studies, such as randomized clinical trials, cohort studies, case-control studies, etc., were found.

### Future lines of research

The scarcity of national studies on the role of podiatrists in the legal and forensic fields leads us to the need for future research. It would be interesting to conduct studies that involve podiatrists in the different aspects of an investigative case or to create valid and useful protocols where podiatrists can freely practice their work. Future research will address objectives such as formulating regression equations based on the length of footprints or the foot itself to estimate the height of both sexes in Spain, as there is no standard formula for estimating height based on foot dimensions in the Spanish population.

## Conclusion

In forensic investigations, the inked footprint technique is the most commonly employed method for estimating stature from footprints, compared to pedigraphy and foot contouring. All three methods demonstrate strong positive correlations between footprint length and individual height. However, despite its prevalence, the inked footprint technique does not currently have superior scientific validation over the other methods due to limited comparative research. Future studies with standardized protocols and larger, diverse populations are necessary to establish the most accurate and reliable technique for practical forensic application.

## Key Points

1. The review showed a correlation between footprint length and a height.
2. The inked footprint method emerged is the most widely used in forensics.
3. Gender should be considered when estimating height based on footprints.
4. Findings are limited to specific populations, requiring further research for broader application.

## Supplementary information

Below is the link to the electronic supplementary material.

Supplementary Material 1 (DOCX 3.75 MB)
