# Supplementary material for: Forensic stature estimation: a systematic review of the correlation between footprints and individual height
Source: Forensic Sci Med Pathol. 2025 Oct 30;22(1):363–71. doi: 10.1007/s12024-025-01110-8 (PMC13133232; doi:10.1007/s12024-025-01110-8)
Supplement: Supplementary file 1 — Supplementary Material 1 (DOCX 3.75 MB) [file 12024_2025_1110_MOESM1_ESM.docx]

**Highlights**

- The review demonstrates a correlation between footprint length and a height.

- The tinted footprint method emerged as the most widely used in forensics.

- Gender should be considered in forensic height estimation based on footprints.

- Findings are limited to specific populations, requiring further research for broader use
